# Adolescent emotional dysregulation and suicide: A review

**DOI:** 10.1097/MD.0000000000043127

**Published:** 2026-09-04

**Authors:** Feifei Xu, Sheng Xu

**Affiliations:** a School of Education, Hanjiang Normal University, Shiyan, China; b Intelligent Laboratory of Child and Adolescent Mental Health and Crisis Intervention of Zhejiang Province, Zhejiang Normal University, Jinhua, China; c School of Humanities and International Education Exchange, Anhui University of Chinese Medicine, Hefei, China.

**Keywords:** cognitive behavior therapy, dialectical behavior therapy, emotional dysregulation, suicide

## Abstract

The purpose of this review is to investigate the relationship between emotional dysregulation and adolescent suicide, as well as advancements in assessment and treatment methods. A literature search was conducted using the CINAHL and MEDLINE databases. The database search occurred during the month of December 2022. This article summarizes the commonly used assessment tools for emotional dysregulation in clinical settings, as well as psychological interventions and research advancements in the field. Emotional dysregulation has a strong correlation with adolescent suicide; however, there is a need for further improvement in assessment tools, particularly focusing on longitudinal observation and assessment. Regarding psychological interventions, it is important to explore more efficient and shorter modalities while including family members to ensure treatment effectiveness.

## 1. Introduction

Suicidal behavior refers to the deliberate or voluntary act of individuals using various means to end their own lives. In 2019, the global age-standardized suicide rate was 9 per 100,000 population, resulting in over 700,000 deaths by suicide.^[1]^ In China, suicide accounts for 3.06% of the total potential years of life lost, ranking as the 10th leading cause of potential years of life lost.^[2]^ Suicidal tendencies often arise in adolescence and peak between the ages of 15 and 24. In China, suicide is the fourth leading cause of death among individuals aged 10 to 19.^[3]^ In recent years, there has been an increasing trend in the occurrence rate of adolescent suicide. Suicidal behavior poses a significant threat to people’s life and health, causing severe harm to individuals, families, and society as a whole, making it a global public health issue. For families, the members who have experienced the suicide of a loved one undergo complex emotional reactions and psychological distress,^[4]^ while also facing an increased risk of suicide themselves.^[5]^ Therefore, interventions and research on adolescent suicide behavior are urgent. To better prevent suicide, it is crucial to understand and identify the risk factors associated with child and adolescent suicide.

Emotion regulation is the process through which individuals manage the onset, intensity, and expression of their emotions. It involves how individuals influence their emotions, when they occur, and how they experience and express them. Emotion regulation is the ability of an individual to respond to the demands of a situation by moderating and adjusting their emotions.^[6]^ It includes various strategies for regulating emotional states, such as self-soothing, reevaluating upsetting events, and inhibiting or initiating emotionally driven behaviors.^[7]^ Individuals with good emotion regulation possess the skills to recognize, monitor, and evaluate their emotional experiences. They are adept at adjusting their emotional responses in a way that aligns with the demands of their goals or situations.^[8]^

Emotion regulation has been linked to the etiology and maintenance of a wide range of psychiatric disorders^[9]^ and has been recognized as a transdiagnostic component underpinning a wide range of psychiatric symptoms.^[10,11]^ There are multiple empirical and theoretical frameworks that have defined, conceptualized, and categorized it. Gross provided the seminal conceptualization by stating that emotions may be modulated at 5 points during emotion production, including contextual choice (deciding whether to avoid or engage with emotionally relevant situations), contextual change (altering components of the physical environment in order to change their effect on emotion, rather than just ignoring the situation), attentional deployment (shifting attention from a specific part of the emotional stimulus to other parts), and cognitive change (recognizing negative thought processes and replacing them with more effective ones), and response modification (directly altering physiological, experiential, or behavioral responses to emotional stimuli).^[12]^

On the other hand, emotion dysregulation refers to an individual’s difficulty in recognizing, accepting, and controlling their own emotions. It involves an inability to cope with and control emotions effectively.^[13]^ Emotion dysregulation leads to the display of emotional responses that are inconsistent with the individual’s goals or situational demands.^[14]^

Currently, there is no consensus on the definition of emotion dysregulation, but most researchers agree that emotion dysregulation refers to the difficulty in using emotion regulation strategies or in choosing appropriate strategies to achieve the goal of emotion management. Although there is no consensus on the definition of emotion dysregulation, most researchers agree that emotion dysregulation is characterized by the following 5 aspects: reduced emotional awareness, inadequate emotional response, intense emotional experience and expression, emotional rigidity, and difficulty in cognitive reappraisal. Reduced emotional awareness, which refers to the ability to recognize and label internal emotional experiences, is considered a major aspect of emotion dysregulation and is often referred to as emotional clarity.^[15,16]^ Emotional under reactivity refers to the tendency of individuals to respond inappropriately to strong emotional stimuli. Emotional reactivity is generally considered to be an individual’s initial, unadjusted response to an emotionally arousing stimulus or event, which can be adjusted by emotion regulation strategies (cognitive reappraisal, attention allocation, etc). Strong emotional experience and expression mainly refers to a strong experience and expression of negative emotions, which the individual manifests as emotional dysregulation. In contrast to emotional flexibility and adaptability, emotional rigidity is mainly characterized by out-of-temporal emotional responses that are dominated by specific emotions.^[17–19]^ Difficulty with cognitive reappraisal refers to the inability to reappraise emotions and assign meaning to them, that is, the inability to reduce the strength of emotional reactions through reappraisal.^[20]^ Most studies have also found that cognitive reappraisal is associated with better mental health and lower levels of psychopathology.^[21,22]^ Conversely, difficulties with cognitive reappraisal play an important role in many psychiatric disorders, such as autism, post-traumatic stress disorder, and borderline personality disorder.^[23–26]^

Research has uncovered a strong correlation between emotional dysregulation and child and adolescent suicide. Mental disorders linked to emotional dysregulation often coexist with suicidal tendencies in young individuals. Investigations have shown associations between mood disorders and key risk factors for suicidal ideation, including depression,^[27]^ anxiety,^[28]^ and impulsivity.^[29]^ Previous research also supports the link between emotional dysregulation and suicidal ideation.^[30]^ Lack of emotion regulation strategies and difficulty recognizing and accepting emotions have been associated with increased suicidal ideation in adolescents,^[31,32]^ whereas aspects of accepting emotions, emotional self-efficacy, and regulating impulses have been associated with decreased suicidal ideation in adolescents.^[33]^ In addition, in adolescents and children, fewer adaptive responses and fewer emotion regulation skills also predict suicidal ideation, planning, and attempts.^[34,35]^ Thus, figuring out what other factors may influence the relationship between emotion dysregulation and suicide risk can provide important insights into understanding and preventing suicidal ideation in adolescents, which is especially important given that suicidal ideation is closely related to suicidal behavior.^[36]^ In summary, the level of emotion regulation is strongly related to the problem of suicide among adolescents.^[37]^ Whereas, when strong negative emotions are experienced as intolerable (denial of commitment), individuals engage in suicidal behavior when they lack perceived strategies to appropriately regulate their emotions and resolve distressing situations (strategies; goals).^[38]^ Despite these challenges, it remains unclear why emotional dysregulation contribute to suicidal behavior in young individuals, which factors are influenced, and the effectiveness and drawbacks of suicide interventions based on emotional dysregulation.

## 2. Methods

### 2.1. Aim

This article provides a comprehensive summary of the relationship between emotional dysregulation and adolescent suicidality, as well as common assessment and psychotherapeutic approaches used in clinical care, and research advances in the field. In addition, the paper highlights future research directions and potential issues to be aware of when intervening with emotional dysregulation during clinical practice.

### 2.2. Searching strategy

A literature search was conducted using the CINAHL and MEDLINE databases. The database search occurred during the month of December 2022. A search was conducted using the key terms (“emotional dysregulation” OR “emotional disorder”) AND “suicide” AND “adolescent” MeSH terms were used in the MEDLINE search, and CINAHL headings were used when searching CINAHL when available. The search limiters included the following: “English language” and “peer- reviewed.”

### 2.3. Inclusion and exclusion criteria of the literature

Inclusion criteria: inclusion of one or more of the following outcomes: suicidal ideation, suicide attempts, and suicide deaths; exploration of the relationship between emotional dysregulation and suicide; and possibility of extracting results from the original literature; exclusion criteria: studies related to suicide such as reviews, case reports, conference abstracts, and research plans; studies in which the study population, study type, interventions, and outcome indicators did not meet the inclusion criteria; studies in which the full text was not available, valid data could not be extracted, or the original data could not be requested; studies with duplicate reports.

## 3. Results

### 3.1. The relationship between emotional dysregulation and suicidal behavior in adolescents

Emotional dysregulation is a crucial factor that underlies various diagnoses and behaviors, making it a transdiagnostic clinical feature.^[39]^ Moreover, it is recognized as a risk factor for suicidal ideation, and it has been proposed that it serves as an underlying mechanism for suicidal thoughts. Researchers have presented a comprehensive framework for emotion regulation, which encompasses multiple dimensions. These dimensions include being aware of, understanding, and accepting emotions, exercising control over impulsive behaviors when faced with unpleasant emotions to engage in goal-directed behaviors, and utilizing adaptive strategies to regulate the intensity and duration of emotional responses.^[40]^ The adolescent stage is particularly significant for comprehending emotion regulation and dysregulation in terms of cognitive, interpersonal, and neurological aspects.^[41]^ Neurologically, there exist differences in the development of the prefrontal cortical areas and the subcortical limbic system during adolescence. The prefrontal cortex, responsible for cognitive control, is not yet fully mature, while the subcortical limbic system, which processes emotional stimuli, is relatively more developed.^[42]^ This discrepancy compromises effective top-down control of the subcortical limbic system, resulting in emotional reactivity and increased risk-taking behaviors commonly observed in adolescence.^[43]^ This imbalance may potentially contribute to the development of emotional dysregulation, as well as suicidal thoughts and behaviors.

The study identified a mediating effect of perceived burden and suicidality acquisition between mood dysregulation and suicide risk among adolescents.^[44]^ The researchers assessed whether mood dysregulation predicted suicidal ideation over a 6-month period. A total of 298 community members at higher suicide risk participated in clinical interviews and completed self-report questionnaires at baseline and 6-month follow-up. The results showed that elevated general mood dysregulation significantly predicted increased suicidal ideation at the 6-month follow-up, even after accounting for initial suicidal ideation, treatment conditions, and negative emotions. These findings support the notion that mood dysregulation is a risk factor for suicidal ideation and provide evidence for its role in suicide attempts.^[45]^ Structural equation modeling was used to explore the relationship between mood dysregulation, internalizing symptoms, non-suicidal self-injury (NSSI), and suicide among 148 undergraduate students. The results indicated that mood dysregulation, specifically through emotional dysregulation, indirectly predicted suicide attempt frequency through internalizing symptoms and NSSI frequency.^[46]^ The researchers also investigated the potential mediating role of mood dysregulation and affective intensity in 125 female patients with bulimia nervosa. The findings suggested that emotional dysregulation mediated the relationship between sexual and emotional abuse and self-harm and suicide attempts.^[47]^ The theory of suicide avoidance may offer insights into the relationship between emotional dysregulation and suicide.^[48]^ According to Baumeister, individuals consider suicide as an escape when they are overwhelmed by intense, aversive negative emotions, and feel incapable of generating adaptive coping strategies. Similarly, the “crying in pain” model proposes that suicide becomes an option when individuals are unable to find alternate ways of escaping emotionally intolerable situations. These intolerable emotions can become problematic if the individual lacks the necessary skills to regulate them. In fact, a recent study found that high emotional reactivity coupled with poor problem-solving skills was associated with a higher risk of recent suicide attempts in adolescents.^[49]^ Haskevitch conducted a longitudinal study involving 547 adolescent patients hospitalized with suicidal ideation and suicide attempts, using the Difficulties in Emotion Regulation Scale (DERS). The study revealed a significant association between the level of emotion regulation skills and suicidal ideation over the past year. Furthermore, adolescents who had attempted suicide prior to hospitalization reported higher levels of emotion dysregulation compared to those with only suicidal ideation. Additionally, a history of past suicide attempts was positively linked to higher levels of emotion dysregulation.^[50]^ Multiple suicide attempters not only exhibit stronger negative emotional states compared to those with a single attempt, but they also demonstrate greater mood dysregulation and impairments in impulse control.^[51]^ In the short term, suicidal behavior may initially alleviate emotional arousal, however, in the long term, it may exacerbate negative affect and become an additional source of stress.

Emotional dysregulation has a significant impact on adolescent suicidal problems, but existing studies have only identified the correlation between emotional dysregulation and suicidal problems, as well as potential mediators and moderators. Additionally, there is no experimental method currently available to verify the actual factors linking emotional dysregulation and suicidal problems. The causes of emotional dysregulation are still uncertain, and future studies can strive to delve deeper into this topic.

### 3.2. Assessment of emotional dysregulation

The biopsychosocial model hypothesizes that genetic, experiential, psychological, clinical symptomatic, sociological, and environmental factors influence an individual’s suicide risk. Any or all of these factors may interact at a given time to cause an individual to commit suicide; and that individual-specific factors mediate this. And assessing mood dysregulation is a complex symptom that crosses diagnostic boundaries and is critical when assessing suicide risk in adolescents.

As mentioned earlier, assessment of emotional dysregulation is commonly available to be measured in psychiatric disorders. The Child Behavior Scale-Disorder Scale (CBCL-DP). CBCL-DP is one of the most commonly used instruments to assess disorders,^[52]^ and disorders have been strongly associated with self-injury and suicidal behaviors in adolescents.^[53]^ In addition, a DP can be generated based on items from the widely used Strengths and Difficulties Questionnaire^[54]^ (SDQ). Like the CBCL-DP, the SDQ-DP contains internalizing and externalizing dimensions, and it is based on items assessing hyperactivity/inattention as well as emotional and behavioral/aggressive problems. The SDQ-DP^[55]^ and the CBCL-DP are highly correlated.^[56]^ In the area of emotional disorders, a number of studies have been conducted with the CBCL-DP. This scale assesses: anxiety, depression, attention deficit/hyperactivity, and aggression. The scale provides a comprehensive picture of an individual’s mood and behavior. Researchers have found that the CBCL-DP is associated with self-injury, suicidal ideation, and suicidal behavior in adolescents compared to the general population.^[57–59]^ Further research found that adolescent and parental emotional dysregulation were uniquely and positively associated with adolescent self-injury and duration of suicidal behavior.^[53]^ A cross-sectional study showed that adolescents with higher CBCL-DP scores in childhood were at increased risk for suicide in adulthood.^[60]^ In a clinical study with a sample of patients presenting with mood symptoms, researchers found that those with the CBCL-DP profile exhibited more severe suicidal ideation compared to those without the CBCL-DP profile.^[61]^ Another study of adolescent patients with concomitant high suicide risk found that levels of emotional and behavioral dysregulation in the first few weeks significantly predicted levels of suicidal ideation a few weeks later. In addition, suicidal ideation in the first few weeks significantly predicted subsequent emotional sensitivity, negative mood intensity, and behavioral disorders. Finally, emotional sensitivity significantly and positively predicted levels of suicidal ideation over the subsequent 6 months. Taken together, these findings suggest that emotional and behavioral dysregulation are associated with levels of suicidal ideation. Both the level of trait dysregulation using the CBCL-DP and the current degree of dysregulation are important in predicting an individual’s suicide risk.

The DERS is an effective and comprehensive self-report scale for assessing difficulties in emotion regulation.^[62]^ The scale consists of 6 sub-scales that were derived from factor analysis: nonacceptance of negative emotions, difficulties engaging in goal-directed behavior when distressed, impulse difficulties, lack of effective emotion regulation strategies, lack of emotional awareness, and lack of emotional clarity. Each item is scored on a 1 to 5 Likert scale, with higher scores indicating greater difficulties. In various clinical and non-clinical samples,^[63,64]^ the DERS has demonstrated high internal consistency and good test–retest reliability. To support its construct validity, total scores and sub-scale scores on the DERS have been found to be related to behavioral, physiological, and neural measures of emotion regulation, as well as various forms of psychopathology such as anxiety disorders, depression, and bipolar disorder.^[46,65]^ Therefore, the DERS may serve as a useful outcome measure for assessing difficulties in the emergency department. Furthermore, some researchers have been using ecological momentary assessment to collect real-time data on emotions and behaviors using mobile electronic devices such as smartphones or smartwatches.^[66]^ Ecological momentary assessment allows for the collection of data at different time points over time and can help researchers examine the intricate associations between changes in emotions and suicidal ideation and attempts. For example, a study that assessed emotions multiple times a day for 6 days found that greater emotional instability was associated with more frequent and severe suicidal ideation.^[67]^ These tools are highly useful in assessing suicide risk and identifying targets for psychological interventions.

The current assessment methods are mostly self-assessment or other-assessment scales, which have a great deal of error and are prone to obtaining erroneous data. In the future, we can try to use emotional pictures or video materials as stimulus materials, and then measure the individual’s physiological signs and neurological indications afterward, in order to further obtain more precise results, which can be used to determine changes in emotion regulation.

### 3.3. Psychotherapy

Emotion dysregulation has been found to be associated with suicidal ideation and behavior in adolescents. Therefore, it is crucial to develop effective clinical interventions that target both emotion dysregulation and suicidal behavior in this age group. Currently, researchers have implemented various interventions to address emotion regulation symptoms in adults, which have also been applied to suicidal children and adolescents. These interventions range from individual-focused to family-focused approaches, as well as comprehensive interventions. However, the research evidence for psychological interventions in adolescents is still relatively limited compared to studies conducted in adults.^[68]^

#### 3.3.1. Cognitive behavior therapy

Cognitive behavior therapy (CBT) has been demonstrated as an effective treatment for depression, but it does not adequately address concerns related to suicide. Consequently, researchers have developed an approach for preventing adolescent suicide called CBT-SP.^[69]^ CBT-SP combines the traditional components of CBT for depression treatment, such as cognitive reframing and behavioral activation, with elements of dialectical behavior therapy (DBT), which is specifically focused on emotion regulation. CBT-SP attributes suicidal behavior to emotional dysregulation and cognitive rigidity.^[70]^ The aim of CBT-SP is to target and address emotional regulation disorders by teaching various emotion regulation skills, including relaxation techniques, positive thinking, emotion recognition, and hope building. The inclusion of family involvement and collaboration is also emphasized, as family factors play a role in the development of regulation disorders. The overall objective of this treatment is to reduce the risk of suicidal behavior in adolescents, accomplished in part by directly addressing emotion regulation disorders. However, the current evidence for the effectiveness of CBT-SP remains limited.^[71]^ Another effective approach in reducing suicidal behavior in adolescents is mood-regulation focused CBT.^[72]^ CBT has also been validated for other disorders. In patients with body dysmorphic disorder, CBT was successful in reducing somatic anxiety and improving emotion regulation. The treatment effect and remission of comorbidity were sustained at a follow-up 3 months later.^[73]^ CBT also improves symptoms in patients with post-traumatic stress disorder, with the ability to regulate emotions playing a role in their recovery.^[74]^ CBT can assist individuals in enhancing their emotion regulation abilities, which can ultimately alleviate symptoms of various disorders. Moreover, it is important to explore the potential of developing CBT into different research programs for specific diseases in order to achieve more effective therapeutic outcomes. This possibility requires further investigation and consideration. In addition the combination of CBT and computer programs can greatly enhance the efficiency of treatment, facilitate its delivery, and greatly reduce patient burden. Future research could attempt to develop programs to alleviate mood and address mood disorders in adolescents in a more targeted manner.

#### 3.3.2. Interpersonal psychotherapy

Interpersonal psychotherapy (IPT) for adolescents^[75]^ is an individual-centered therapeutic intervention that has been proven effective in treating adolescents with self-harming thoughts and behaviors.^[76]^ Alongside addressing the impact of adolescent relationships on psychiatric symptoms and overall functioning, IPT for adolescents also focuses on enhancing emotion regulation skills. These skills include emotion education and awareness, emotion monitoring, and emotion expression.^[77]^ Previous studies conducted with adults have shown the effectiveness of brief, focused therapy for patients at higher risk for suicide. For instance, Gysin-Maillart et al conducted a study comparing 2 groups of adults who had attempted suicide and presented to the emergency room. Both groups received routine treatment, which included inpatient hospitalization, day patient care, and individual outpatient care as deemed necessary by the responsible clinician. The study group received 3 additional sessions, each lasting 60 to 90 minutes, specifically focusing on suicide attempts. The results revealed a significant reduction in suicide attempts among the study condition group compared to the control group.^[78]^ In another study that examined patients with depression, including groups receiving CBT, IPT, medication, and placebo treatment, IPT and medication were found to be particularly effective in reducing suicidal ideation when compared to the placebo group.^[79]^ IPT can be implemented in a wider range of environments (e.g., schools) to solve problems in an interpersonal setting, achieve emotional stability, and then achieve a better psychological state. Future research can implement IPT in student classes to achieve the goal of group therapy, but the effectiveness of group therapy needs to be verified by more experiments.

#### 3.3.3. Dialectical behavior therapy

DBT is a comprehensive treatment approach that addresses various aspects, including participation in treatment, reduction of self-harm and suicide attempts, and the acquisition of skills that improve emotional regulation, pain tolerance, and overall life satisfaction.^[80]^ DBT consists of 5 sets of therapeutic strategies used across all treatment modalities: dialectical strategies; core strategies, such as validation and problem-solving, which incorporate standard CBT techniques like behavioral assessment, psychoeducation, therapeutic rationale orientation, contingency management, skills training, exposure, and cognitive modification strategies; communication strategies, involving nonconfrontational and reciprocal communication styles; case management strategies, including consultation with the patient, environmental interventions, and consultation with the therapist; and structural strategies, focused on goal orientation within sessions and the initiation and closure of treatment. Therapists must employ a balanced approach, blending strategies that range from rapid juxtaposition of change and acceptance to the use of communication styles that are both nonconfrontational and warmly responsive, to effectively address the diverse needs of their clients. This adaptability is crucial in supporting ongoing progress and managing the fluctuating challenges that clients with disorders may present.^[81]^

Research has demonstrated the efficacy of DBT with adults, showing low dropout rates and significant reductions in suicide attempts and NSSI.^[82]^ DBT targets emotional, interpersonal, and behavioral dysregulation that contribute to self-injurious thoughts and behaviors.^[83,84]^ In a recent randomized controlled trial involving self-harming adolescents, DBT was found to be highly effective in reducing self-harm and suicidal ideation compared to conventional treatment, with maintained effects at a 1-year follow-up.^[85,86]^ DBT also remains effective for adolescents at higher risk for suicide attempts.^[87]^ The correlation between DBT and remission of self-harm during follow-up was mediated by improved emotion regulation in adolescents, who reported fewer substance abuse issues, externalizing behaviors, and overall problems 6 months posttreatment and throughout the 12-month follow-up period. Therefore, these findings emphasize the significance of emotion regulation as a treatment goal to reduce self-harm, and suggest that DBT also offers advantages in addressing substance abuse and externalizing behaviors.^[88]^

Furthermore, it is crucial to acknowledge the influence of family relationships on an individual’s emotional state.^[89]^ As a result, it is reasonable for researchers to incorporate family relationship regulation as a treatment goal or to focus on such relationships in their interventions.^[90]^ For instance, attachment-based family therapy is grounded in attachment theory and focuses on teaching interpersonal and emotion regulation skills that ultimately enhance the quality of the parent–adolescent relationship and foster relational security in adolescents, enabling them to confront and cope with difficult issues. Numerous other family-focused interventions, as well as interventions that combine individual therapy with family components, have been developed and tested, and their effectiveness has been extensively reviewed elsewhere.^[91]^ Notably, a meta-analysis and review of interventions for suicidal behavior in adolescents has revealed that those with a family component exhibit stronger evidence of efficacy in the treatment of suicide and self-harm.^[92]^ In conclusion, when selecting interventions for adolescent suicidality and emotional dysregulation, it is important to consider the inclusion of family information, and given the lengthy durations of traditional psychological interventions, future treatments should also explore the possibility of offering brief treatment options. Future research needs to further elucidate the mechanisms that contribute to the recovery of adolescents at high risk for suicidal/self-destructive behaviors, clarify strategies to optimize treatment to prevent the risk of persistence and/or relapse, and ultimately prevent the tragedy of suicide deaths.

## 4. Conclusions

A growing body of evidence suggests a strong relationship between emotional dysregulation and suicide in adolescents. This relationship extends across the psychiatric diagnostic field and increases the risk of self-harm. Currently, suicide risk assessments for children and adolescents do not include structured assessments of emotional dysregulation and externalizing behaviors. Despite incorporating information about an individual’s clinical symptoms, environment, social factors, and biological risk factors, existing suicide risk assessments do not accurately predict the occurrence of suicidal behavior. While these assessments can predict that an individual’s risk of suicide will increase at a certain point in time, they cannot predict who will commit suicide. The diagnosis given to an individual may influence their response to a stressor, either by increasing the impact of a specific stressor or decreasing their ability to act on consistent suicidal impulses under stress. However, it does not determine who will ultimately commit suicide. Instead, a person’s ability to tolerate negative emotions and their coping skills and strategies determine their response to suicidal thoughts. Research on intervention treatments indicates that effective interventions for emotion regulation disorders in children and adolescents, such as DBT, are crucial. CBT and family-based interventions have also shown promise for youth. Despite the effectiveness of psychotherapy, its role remains limited. Emotional dysregulation plays a significant role in moving individuals from suicidal thoughts to action. Future research could include assessing emotional dysregulation in clinical studies to increase awareness and develop targeted interventions to influence suicide trends in adolescents.

## Author contributions

**Conceptualization:** Feifei Xu, Sheng Xu.

**Formal analysis:** Feifei Xu, Sheng Xu.

**Funding acquisition:** Sheng Xu.

**Investigation:** Feifei Xu, Sheng Xu.

**Methodology:** Feifei Xu.

**Resources:** Feifei Xu, Sheng Xu.

**Software:** Sheng Xu.

**Validation:** Feifei Xu, Sheng Xu.

**Visualization:** Feifei Xu, Sheng Xu.

**Writing – original draft:** Feifei Xu.

**Writing – review & editing:** Sheng Xu.

## References

[1] WHO. Suicide worldwide in 2019. https://www.who.int/publications-detail-redirect/9789240026643. Accessed February 17, 2025.

[2] Sun L Zhang J. Potential years of life lost due to suicide in China, 2006-2010. Public Health. 2015;129:555–60. 25769349 10.1016/j.puhe.2015.02.012 PMC4442732

[3] Xu RB Wen B Song Y. The change in mortality and major causes of death among Chinese adolescents from 1990 to 2016. Chi J Prev Med. 2018;52:802–8. 10.3760/cma.j.issn.0253-9624.2018.08.006 30107713

[4] Cerel J Jordan JR Duberstein PR. The impact of suicide on the family. Crisis. 2008;29:38–44. 18389644 10.1027/0227-5910.29.1.38

[5] Pitman A Osborn D King M Erlangsen A. Effects of suicide bereavement on mental health and suicide risk. Lancet Psychiatry. 2014;1:86–94. 26360405 10.1016/S2215-0366(14)70224-X

[6] Cole PM Michel MK Teti LO. The development of emotion regulation and dysregulation: a clinical perspective. Monogr Soc Res Child Dev. 1994;59:73–100. 7984169

[7] Eisenberg N Gershoff ET Fabes RA. Mothers’ emotional expressivity and children’s behavior problems and social competence: mediation through children’s regulation. Dev Psychol. 2001;37:475–90. 11444484 10.1037//0012-1649.37.4.475

[8] Eisenberg N Morris AS. Children’s emotion-related regulation. Adv Child Dev Behav. 2002;30:189–229. 12402675

[9] Gross JJ. Emotion regulation: affective, cognitive, and social consequences. Psychophysiology. 2002;39:281–91. 12212647 10.1017/s0048577201393198

[10] Cole PM Martin SE Dennis TA. Emotion regulation as a scientific construct: methodological challenges and directions for child development research. Child Dev. 2004;75:317–33. 15056186 10.1111/j.1467-8624.2004.00673.x

[11] Barlow DH Allen LB Choate ML. Toward a unified treatment for emotional disorders. Behav Ther. 2004;35:205–30. 10.1016/j.beth.2016.11.005 27993336

[12] Gross JJ. The emerging field of emotion regulation: an integrative review. Rev General Psychol. 1998;2:271–99.

[13] Gratz KL Roemer L. Multidimensional assessment of emotion regulation and dysregulation: development, factor structure, and initial validation of the difficulties in emotion regulation scale (vol 26, pg 41, 2004). J Psychopathol Behav Assess. 2008;30:315–315.

[14] Zeman J Cassano M Perry-Parrish C Stegall S. Emotion regulation in children and adolescents. J Dev Behav Pediatr. 2006;27:155–68. 16682883 10.1097/00004703-200604000-00014

[15] Sifneos PE. The prevalence of “alexithymic” characteristics in psychosomatic patients. Psychother Psychosom. 1973;22:255–62. 4770536 10.1159/000286529

[16] Penza-Clyve S Zeman J. Initial validation of the Emotion Expression Scale for Children (EESC). J Clin Child Adolesc Psychol. 2002;31:540–7. 12402572 10.1207/S15374424JCCP3104_12

[17] Thompson RA. Methods and measures in developmental emotions research: some assembly required. J Exp Child Psychol. 2011;110:275–85. 21592492 10.1016/j.jecp.2011.04.007

[18] Bonanno GA Papa A Lalande K Westphal M Coifman K. The importance of being flexible: the ability to both enhance and suppress emotional expression predicts long-term adjustment. Psychol Sci. 2004;15:482–7. 15200633 10.1111/j.0956-7976.2004.00705.x

[19] Paivio SC Greenberg LS. Introduction: Treating emotion regulation problems. J Clin Psychol. 2001;57:153–5. 11180143 10.1002/1097-4679(200102)57:2<153::aid-jclp2>3.0.co;2-f

[20] Gross JJ. Emotion regulation: affective, cognitive, and social consequences. Psychophysiology. 2002;39:281–91. 12212647 10.1017/s0048577201393198

[21] Gross JJ John OP. Individual differences in two emotion regulation processes: implications for affect, relationships, and well-being. J Pers Soc Psychol. 2003;85:348–62. 12916575 10.1037/0022-3514.85.2.348

[22] Eftekhari A Zoellner LA Vigil SA. Patterns of emotion regulation and psychopathology. Anxiety Stress Coping. 2009;22:571–86. 19381989 10.1080/10615800802179860 PMC3234115

[23] Schulze L Domes G Krüger A. Neuronal correlates of cognitive reappraisal in borderline patients with affective instability. Biol Psychiatry. 2011;69:564–73. 21195392 10.1016/j.biopsych.2010.10.025

[24] Ehlers A Maercker A Boos A. Posttraumatic stress disorder following political imprisonment: the role of mental defeat, alienation, and perceived permanent change. J Abnorm Psychol. 2000;109:45–55. 10740935

[25] Ehlers A Mayou RA Bryant B. Psychological predictors of chronic posttraumatic stress disorder after motor vehicle accidents. J Abnorm Psychol. 1998;107:508–19. 9715585 10.1037//0021-843x.107.3.508

[26] Samson AC Hardan AY Lee IA Phillips JM Gross JJ. Maladaptive Behavior in autism spectrum disorder: the role of emotion experience and emotion regulation. J Autism Dev Disord. 2015;45:3424–32. 25711546 10.1007/s10803-015-2388-7

[27] May AM Klonsky ED Klein DN. Predicting future suicide attempts among depressed suicide ideators: a 10-year longitudinal study. J Psychiatr Res. 2012;46:946–52. 22575331 10.1016/j.jpsychires.2012.04.009 PMC3372684

[28] Sareen J Cox BJ Afifi TO. Anxiety disorders and risk for suicidal ideation and suicide attempts: a population-based longitudinal study of adults. Arch Gen Psychiatry. 2005;62:1249–57. 16275812 10.1001/archpsyc.62.11.1249

[29] Baca-García E Diaz-Sastre C Basurte E. A prospective study of the paradoxical relationship between impulsivity and lethality of suicide attempts. J Clin Psychiatry. 2001;62:560–4. 11488369 10.4088/jcp.v62n07a11

[30] Tamás Z Kovacs M Gentzler AL. The relations of temperament and emotion self-regulation with suicidal behaviors in a clinical sample of depressed children in Hungary. J Abnorm Child Psychol. 2007;35:640–52. 17530394 10.1007/s10802-007-9119-2

[31] Hatkevich C Penner F Sharp C. Difficulties in emotion regulation and suicide ideation and attempt in adolescent inpatients. Psychiatry Res. 2019;271:230–8. 30502560 10.1016/j.psychres.2018.11.038

[32] Wolff JC Davis S Liu RT. Trajectories of suicidal ideation among adolescents following psychiatric hospitalization. J Abnorm Child Psychol. 2018;46:355–63. 28349306 10.1007/s10802-017-0293-6 PMC5617752

[33] Valois RF Zullig KJ Hunter AA. Association between adolescent suicide ideation, suicide attempts and emotional self-efficacy. J Child Fam Stud. 2015;24:237–48.

[34] Brausch AM Woods SE. Emotion regulation deficits and nonsuicidal self-injury prospectively predict suicide ideation in adolescents. Suicide Life Threat Behav. 2019;49:868–80. 29900570 10.1111/sltb.12478 PMC6294703

[35] Tamás Z Kovacs M Gentzler AL. The relations of temperament and emotion self-regulation with suicidal behaviors in a clinical sample of depressed children in Hungary. J Abnorm Child Psychol. 2007;35:640–52. 17530394 10.1007/s10802-007-9119-2

[36] Glenn CR Nock MK. Improving the short-term prediction of suicidal behavior. Am J Prev Med. 2014;47:S176–80. 25145736 10.1016/j.amepre.2014.06.004 PMC5258198

[37] Rogante E Cifrodelli M Sarubbi S. The role of emotion dysregulation in understanding suicide risk: a systematic review of the literature. Healthcare (Basel). 2024;12:169. 38255058 10.3390/healthcare12020169 PMC10815449

[38] Rasmussen SA Fraser L Gotz M. Elaborating the cry of pain model of suicidality: testing a psychological model in a sample of first-time and repeat self-harm patients. Br J Clin Psychol. 2010;49:15–30. 19302734 10.1348/014466509X415735

[39] Berking M Wupperman P Reichardt A Pejic T Dippel A Znoj H. Emotion-regulation skills as a treatment target in psychotherapy. Behav Res Ther. 2008;46:1230–7. 18835479 10.1016/j.brat.2008.08.005

[40] Gratz KL Roemer L. Multidimensional assessment of emotion regulation and dysregulation: development, factor structure, and initial validation of the difficulties in emotion regulation scale (vol 26, pg 41, 2004). J Psychopathol Behav Assess. 2008;30:315–315.

[41] Spear LP. The adolescent brain and age-related behavioral manifestations. Neurosci Biobehav Rev. 2000;24:417–63. 10817843 10.1016/s0149-7634(00)00014-2

[42] Ernst M Nelson EE Jazbec S. Amygdala and nucleus accumbens in responses to receipt and omission of gains in adults and adolescents. Neuroimage. 2005;25:1279–91. 15850746 10.1016/j.neuroimage.2004.12.038

[43] Romer D. Adolescent risk taking, impulsivity, and brain development: implications for prevention. Dev Psychobiol. 2010;52:263–76. 20175097 10.1002/dev.20442 PMC3445337

[44] Eaddy M Zullo L Horton SE. A theory-driven investigation of the association between emotion dysregulation and suicide risk in a clinical adolescent sample. Suicide Life Threat Behav. 2019;49:928–40. 29745436 10.1111/sltb.12472

[45] Raudales AM Short NA Schmidt NB. Emotion dysregulation as a prospective predictor of suicidal ideation in an at-risk mixed clinical sample. Arch Suicide Res. 2020;24:S310–22. 30955483 10.1080/13811118.2019.1598526

[46] Kranzler A Fehling KB Anestis MD Selby EA. Emotional dysregulation, internalizing symptoms, and self-injurious and suicidal behavior: Structural equation modeling analysis. Death Stud. 2016;40:358–66. 26808092 10.1080/07481187.2016.1145156

[47] Gordon KH Simonich H Wonderlich SA. Emotion dysregulation and affective intensity mediate the relationship between childhood abuse and suicide-related behaviors among women with bulimia nervosa. Suicide Life Threat Behav. 2016;46:79–87. 26052753 10.1111/sltb.12172 PMC8711241

[48] Baumeister RF. Suicide as escape from self. Psychol Rev. 1990;97:90–113. 2408091 10.1037/0033-295x.97.1.90

[49] Dour HJ Cha CB Nock MK. Evidence for an emotion-cognition interaction in the statistical prediction of suicide attempts. Behav Res Ther. 2011;49:294–8. 21353203 10.1016/j.brat.2011.01.010

[50] Zlotnick C Donaldson D Spirito A Pearlstein T. Affect regulation and suicide attempts in adolescent inpatients. J Am Acad Child Adolesc Psychiatry. 1997;36:793–8. 9183134 10.1097/00004583-199706000-00016

[51] Esposito C Spirito A Boergers J Donaldson D. Affective, behavioral, and cognitive functioning in adolescents with multiple suicide attempts. Suicide Life Threat Behav. 2003;33:389–99. 14695054 10.1521/suli.33.4.389.25231

[52] Biederman J Petty CR Day H. Severity of the aggression/anxiety-depression/attention child behavior checklist profile discriminates between different levels of deficits in emotional regulation in youth with attention-deficit hyperactivity disorder. J Dev Behav Pediatr. 2012;33:236–43. 22278125 10.1097/DBP.0b013e3182475267 PMC3319866

[53] Deutz MHF Geeraerts SB van Baar AL Deković M Prinzie P. The Dysregulation Profile in middle childhood and adolescence across reporters: factor structure, measurement invariance, and links with self-harm and suicidal ideation. Eur Child Adolesc Psychiatry. 2016;25:431–42. 26226917 10.1007/s00787-015-0745-x PMC4820491

[54] Goodman R. The strengths and difficulties questionnaire: a research note. J Child Psychol Psychiatry. 1997;38:581–6. 9255702 10.1111/j.1469-7610.1997.tb01545.x

[55] Deutz MHF Shi Q Vossen HGM. Evaluation of the Strengths and Difficulties Questionnaire-Dysregulation Profile (SDQ-DP). Psychol Assess. 2018;30:1174–85. 29927304 10.1037/pas0000564 PMC6107405

[56] Gratz KL Roemer L. Multidimensional assessment of emotion regulation and dysregulation: development, factor structure, and initial validation of the difficulties in emotion regulation scale (vol 26, pg 41, 2004). J Psychopathol Behav Assess. 2008;30:315–315.

[57] Volk HE Todd RD. Does the child behavior checklist juvenile bipolar disorder phenotype identify bipolar disorder? Biol Psychiatry. 2007;62:115–20. 16950211 10.1016/j.biopsych.2006.05.036

[58] Ayer L Althoff R Ivanova M. Child Behavior Checklist Juvenile Bipolar Disorder (CBCL-JBD) and CBCL Posttraumatic Stress Problems (CBCL-PTSP) scales are measures of a single dysregulatory syndrome. J Child Psychol Psychiatry. 2009;50:1291–300. 19486226 10.1111/j.1469-7610.2009.02089.x

[59] Meyer SE Carlson GA Youngstrom E. Long-term outcomes of youth who manifested the CBCL-Pediatric Bipolar Disorder phenotype during childhood and/or adolescence. J Affect Disord. 2009;113:227–35. 18632161 10.1016/j.jad.2008.05.024

[60] Selby EA Yen S Spirito A. Time varying prediction of thoughts of death and suicidal ideation in adolescents: weekly ratings over 6-month follow-up. J Clin Child Adolesc Psychol. 2013;42:481–95. 23148530 10.1080/15374416.2012.736356 PMC3584233

[61] Mbekou V Gignac M MacNeil S Mackay P Renaud J. The CBCL dysregulated profile: an indicator of pediatric bipolar disorder or of psychopathology severity? J Affect Disord. 2014;155:299–302. 24230916 10.1016/j.jad.2013.10.033

[62] Gratz KL Roemer L. Multidimensional assessment of emotion regulation and dysregulation: development, factor structure, and initial validation of the difficulties in emotion regulation scale (vol 26, pg 41, 2004). J Psychopathol Behav Assess. 2004;30:315–315.

[63] Fowler JC Clapp JD Madan A Allen JG Oldham JM Frueh BC. Emotion dysregulation as a cross-cutting target for inpatient psychiatric intervention. J Affect Disord. 2016;206:224–31. 27479535 10.1016/j.jad.2016.07.043

[64] Hallion LS Steinman SA Tolin DF Diefenbach GJ. Psychometric properties of the Difficulties in Emotion Regulation Scale (DERS) and its short forms in adults with emotional disorders. Front Psychol. 2018;9:539. 29725312 10.3389/fpsyg.2018.00539 PMC5917244

[65] Vasilev CA Crowell SE Beauchaine TP Mead HK Gatzke-Kopp LM. Correspondence between physiological and self-report measures of emotion dysregulation: a longitudinal investigation of youth with and without psychopathology. J Child Psychol Psychiatry. 2009;50:1357–64. 19811585 10.1111/j.1469-7610.2009.02172.x

[66] Ebner-Priemer UW Trull TJ. Ecological momentary assessment of mood disorders and mood dysregulation. Psychol Assess. 2009;21:463–75. 19947781 10.1037/a0017075

[67] Palmier-Claus JE Taylor PJ Gooding P Dunn G Lewis SW. Affective variability predicts suicidal ideation in individuals at ultra-high risk of developing psychosis: an experience sampling study. Br J Clin Psychol. 2012;51:72–83. 22268542 10.1111/j.2044-8260.2011.02013.x

[68] Calati R Courtet P. Is psychotherapy effective for reducing suicide attempt and non-suicidal self-injury rates? Meta-analysis and meta-regression of literature data. J Psychiatr Res. 2016;79:8–20. 27128172 10.1016/j.jpsychires.2016.04.003

[69] Stanley B Brown G Brent DA. Cognitive-Behavioral Therapy for Suicide Prevention (CBT-SP): treatment model, feasibility, and acceptability. J Am Acad Child Adolesc Psychiatry. 2009;48:1005–13. 19730273 10.1097/CHI.0b013e3181b5dbfe PMC2888910

[70] Bryan CJ. Cognitive behavioral therapy for suicide prevention (CBT-SP): Implications for meeting standard of care expectations with suicidal patients. Behav Sci Law. 2019;37:247–58. 31119794 10.1002/bsl.2411

[71] Alavi A Sharifi B Ghanizadeh A Dehbozorgi G. Effectiveness of cognitive-behavioral therapy in decreasing suicidal ideation and hopelessness of the adolescents with previous suicidal attempts. Iran J Pediatr. 2013;23:467–72. 24427502 PMC3883378

[72] Högberg G Hällström T. Mood regulation focused CBT based on memory reconsolidation, reduced suicidal ideation and depression in youth in a randomised controlled study. Int J Environ Res Public Health. 2018;15:921. 29734740 10.3390/ijerph15050921 PMC5981960

[73] Mohajerin B Bakhtiyar M Olesnycky OS Dolatshahi B Motabi F. Application of a transdiagnostic treatment for emotional disorders to body dysmorphic disorder: a randomized controlled trial. J Affect Disord. 2019;245:637–44. 30445389 10.1016/j.jad.2018.11.058

[74] Hinton DE Hofmann SG Pollack MH Otto MW. Mechanisms of efficacy of CBT for Cambodian refugees with PTSD: improvement in emotion regulation and orthostatic blood pressure response. CNS Neurosci Ther. 2009;15:255–63. 19691545 10.1111/j.1755-5949.2009.00100.x PMC6494047

[75] Mufson L Moreau D Weissman MM Wickramaratne P Martin J Samoilov A. Modification of interpersonal psychotherapy with depressed adolescents (IPT-A): phase I and II studies. J Am Acad Child Adolesc Psychiatry. 1994;33:695–705. 8056733 10.1097/00004583-199406000-00011

[76] Haruvi Catalan L Levis Frenk M Adini Spigelman E. Ultra-Brief crisis IPT-A based intervention for suicidal children and adolescents (IPT-A-SCI) pilot study results. Front Psychiatry. 2020;11:553422. 33362595 10.3389/fpsyt.2020.553422 PMC7755882

[77] Tang T-C Jou S-H Ko C-H Huang S-Y Yen C-F. Randomized study of school-based intensive interpersonal psychotherapy for depressed adolescents with suicidal risk and parasuicide behaviors. Psychiatry Clin Neurosci. 2009;63:463–70. 19531111 10.1111/j.1440-1819.2009.01991.x

[78] Gysin-Maillart A Schwab S Soravia L Megert M Michel K. A novel brief therapy for patients who attempt suicide: a 24-months follow-up randomized controlled study of the attempted suicide short intervention program (ASSIP). PLoS Med. 2016;13:e1001968. 26930055 10.1371/journal.pmed.1001968 PMC4773217

[79] Weitz E Hollon SD Kerkhof A Cuijpers P. Do depression treatments reduce suicidal ideation? The effects of CBT, IPT, pharmacotherapy, and placebo on suicidality. J Affect Disord. 2014;167:98–103. 24953481 10.1016/j.jad.2014.05.036

[80] Groves S Backer HS van den Bosch W Miller A. Dialectical behaviour therapy with adolescents. Child Adolesc Ment Health. 2012;17:65–75. 32847295 10.1111/j.1475-3588.2011.00611.x

[81] DeCou CR Comtois KA Landes SJ. Dialectical behavior therapy is effective for the treatment of suicidal behavior: a meta-analysis. Behav Ther. 2019;50:60–72. 30661567 10.1016/j.beth.2018.03.009

[82] Linehan MM Korslund KE Harned MS. Dialectical behavior therapy for high suicide risk in individuals with borderline personality disorder: a randomized clinical trial and component analysis. JAMA Psychiatry. 2015;72:475–82. 25806661 10.1001/jamapsychiatry.2014.3039

[83] Shelton D Kesten K Zhang W Trestman R. Impact of a Dialectic Behavior Therapy-Corrections Modified (DBT-CM) upon behaviorally challenged incarcerated male adolescents. J Child Adolesc Psychiatr Nurs. 2011;24:105–13. 21501287 10.1111/j.1744-6171.2011.00275.x PMC3080237

[84] Klein DA Miller AL. Dialectical behavior therapy for suicidal adolescents with borderline personality disorder. Child Adolesc Psychiatr Clin N Am. 2011;20:205–16. 21440851 10.1016/j.chc.2011.01.001

[85] Mehlum L Tørmoen AJ Ramberg M. Dialectical behavior therapy for adolescents with repeated suicidal and self-harming behavior: a randomized trial. J Am Acad Child Adolesc Psychiatry. 2014;53:1082–91. 25245352 10.1016/j.jaac.2014.07.003

[86] Mehlum L Ramberg M Tørmoen AJ. Dialectical behavior therapy compared with enhanced usual care for adolescents with repeated suicidal and self-harming behavior: outcomes over a one-year follow-up. J Am Acad Child Adolesc Psychiatry. 2016;55:295–300. 27015720 10.1016/j.jaac.2016.01.005

[87] McCauley E Berk MS Asarnow JR. Efficacy of dialectical behavior therapy for adolescents at high risk for suicide: a randomized clinical trial. JAMA Psychiatry. 2018;75:777–85. 29926087 10.1001/jamapsychiatry.2018.1109 PMC6584278

[88] Asarnow JR Berk MS Bedics J. Dialectical behavior therapy for suicidal self-harming youth: emotion regulation, mechanisms, and mediators. J Am Acad Child Adolesc Psychiatry. 2021;60:1105–15.e4. 33539915 10.1016/j.jaac.2021.01.016

[89] Morris AS Silk JS Steinberg L Myers SS Robinson LR. The role of the family context in the development of emotion regulation. Soc Dev. 2007;16:361–88. 19756175 10.1111/j.1467-9507.2007.00389.x PMC2743505

[90] Frey LM Hunt QA. Treatment for suicidal thoughts and behavior: a review of family-based interventions. J Marital Fam Ther. 2018;44:107–24. 28394014 10.1111/jmft.12234

[91] Ougrin D Tranah T Stahl D Moran P Asarnow JR. Therapeutic interventions for suicide attempts and self-harm in adolescents: systematic review and meta-analysis. J Am Acad Child Adolesc Psychiatry. 2015;54:97–107.e2. 25617250 10.1016/j.jaac.2014.10.009

[92] Asarnow JR Mehlum L. Practitioner Review: treatment for suicidal and self-harming adolescents – advances in suicide prevention care. J Child Psychol Psychiatry. 2019;60:1046–54. 31512763 10.1111/jcpp.13130 PMC6880954

